# Decreased prevalence of the *Plasmodium falciparum Pfcrt* K76T and *Pfmdr1* and N86Y mutations post-chloroquine treatment withdrawal in Katete District, Eastern Zambia

**DOI:** 10.1186/s12936-021-03859-z

**Published:** 2021-07-28

**Authors:** Mwenda C. Mulenga, Lungowe Sitali, Ilinca I. Ciubotariu, Moonga B. Hawela, Busiku Hamainza, James Chipeta, Sungano Mharakurwa

**Affiliations:** 1 PATH Malaria Control and Elimination Partnership in Africa (MACEPA), National Malaria Elimination Centre, Ministry of Health, Chainama Grounds, Lusaka, Zambia; 2 School of Health Sciences, Biomedical Sciences Department, Ridgeway campus, Lusaka, Zambia; 3 School of Medicine, University Teaching Hospital Malaria Research Unit (SMUTH-MRU), Lusaka, Zambia; 4 Department of Biological Sciences, Purdue University, West Lafayette, IN USA; 5 Malaria Elimination Centre, Ministry of Health, Chainama Hospital and College Grounds, Lusaka, Zambia; 6 College of Health, Agriculture and Natural Sciences, Africa University, Mutare, Zimbabwe

**Keywords:** Chloroquine resistance, Chloroquine sensitivity, Malaria, Mutation, Genotypes

## Abstract

**Background:**

In 2002, Zambia withdrew chloroquine as first-line treatment for *Plasmodium falciparum* malaria due to increased treatment failure and worldwide spread of chloroquine resistance. The artemisinin combination regimen, artemether–lumefantrine, replaced chloroquine (CQ) as first choice malaria treatment. The present study determined the prevalence of CQ resistance molecular markers in the *Pfcrt* and *Pfmdr1* genes in Eastern Zambia at 9 and 13 years after the removal of drug pressure.

**Methods:**

Samples collected from Katete District during the drug therapeutic efficacy assessments conducted in 2012 and 2016 were assayed by polymerase chain reaction (PCR) and restriction fragment length polymorphisms (RFLP) to determine the prevalence of genetic mutations, K76T on the *Pfcrt* gene and N86Y on the *Pfmdr1* gene. A total of 204 *P. falciparum*-positive DBS samples collected at these two time points were further analysed.

**Results:**

Among the samples analysed for *Pfcrt* K76T and *Pfmdr1* N86Y in the present study, 112 (82.4%) *P. falciparum-*infected samples collected in 2012 were successfully amplified for *Pfcrt* and 94 (69.1%) for *Pfmdr1*, while 69 (65.7%) and 72 (68.6%) samples from 2016 were successfully amplified for *Pfcrt* and *Pfmdr1*, respectively. In 2012, the prevalence of *Pfcrt* 76K (sensitive) was 97.3%, 76T (resistant) was 1.8%, and 0.8% had both 76K and 76T codons (mixed). Similarly in 2012, the prevalence of *Pfmdr1* 86N (sensitive) was 97.9% and 86Y (resistant) was 2.1%. In the 2016 samples, the prevalence of the respective samples was 100% *Pfcrt* 76K and *Pfmdr1* 86N.

**Conclusion:**

This study shows that there was a complete recovery of chloroquine-sensitive parasites by 2016 in Katete District, Eastern Zambia, 13 years following the withdrawal of CQ in the country. These findings add to the body of evidence for a fitness cost in CQ-resistant *P. falciparum* in Zambia and elsewhere. Further studies are recommended to monitor resistance countrywide and explore the feasibility of integration of the former best anti-malarial in combination therapy in the future.

## Background

Malaria continues to be endemic throughout Zambia and remains a significant public health concern. Over the past two decades, significant strides have been made in reducing overall malaria mortality and morbidity in the country. Multiple successes such as a 50% reduction in malaria cases and deaths between 2000 and 2015 have been observed, largely due to scale-up of vector control interventions and malaria treatment as recommended by the World Health Organization (WHO) [1]. Additionally, the incidence of malaria in Zambia decreased from 409 cases per 1000 in 2014 to 335 cases per 1000 in 2015 [2]. Based on these and other measures of recent success, the National Malaria Elimination Programme pledged to eliminate malaria in Zambia through the continued use of these effective vector control interventions, in combination with sustained and prompt case management, health promotion and surveillance [3, 4]. However, development of drug resistance in the de facto malaria parasite in Zambia, *Plasmodium falciparum*, continues to threaten these successes. This was evidenced in the country, where anti-malarial resistance developed to chloroquine and sulfadoxine–pyrimethamine [5]. Following recommendations set forth by the WHO after widespread treatment failures and the spread of chloroquine (CQ) resistance were observed, Zambia withdrew CQ as the first-line drug for uncomplicated falciparum malaria and replaced it with artemether-lumefantrine (AL), an artemisinin-based combination therapy (ACT) regimen in 2002 [6]. AL is recommended for the treatment of uncomplicated cases as it results in the rapid reduction of malaria parasite load [7].

Early studies have suggested that a single mutation which results in the replacement of lysine (K) by threonine (T) at amino acid codon position 76 of the *P. falciparum* CQ-resistant transporter *(Pfcrt)* gene (chromosome 7), is responsible for CQ resistance [8]. This K76T mutation was established as the most useful prognostic marker for treatment failure [9]. Moreover, this point mutation has been linked to CQ resistance in isolates collected worldwide. Furthermore, there is another point mutation N86Y of the *P. falciparum* multi drug-resistant gene 1 (*Pfmdr1*) located on chromosome 5 that also appears to play a role in CQ resistance [8]. Some studies have shown that there is an association between the *Pfcrt* K76T mutation and the mutation on amino acid codon 86 of the *Pfmdr1* gene in CQ resistance, although others refute the association [10, 11].

CQ efficacy is thought to lie in its ability to interrupt haematin detoxification in malaria parasites as they grow in their host blood cells. Specifically, CQ acts against the trophozoite and schizont stages of the *P. falciparum* parasites. Haematin is released in large amounts as the parasite consumes and digests haemoglobin in its digestive food vacuole. Haematin normally is detoxified by polymerization into innocuous crystals of haemozoin [12]. CQ does not allow for the proper detoxification of haematin by forming a drug: haematin complex. The disruption of the haematin detoxification process by quinolines has destructive consequences for the parasites [12].

In recent years, most malaria-endemic countries, including Zambia, have reported the re-emergence of CQ susceptible parasites in regions where there has been a sustained withdrawal of CQ, which has mostly resulted from the re-expansion of the wild type after the removal of drug pressure [13–18]. The present study was conducted to ascertain the prevalence of the *Pfcrt* K76T and *Pfmdr1* N86Y mutations in *P. falciparum* parasites in Katete District, in Eastern Province, Zambia after the withdrawal of CQ as first-line treatment.

## Methods

### Study site and design

**Fig. 1:**
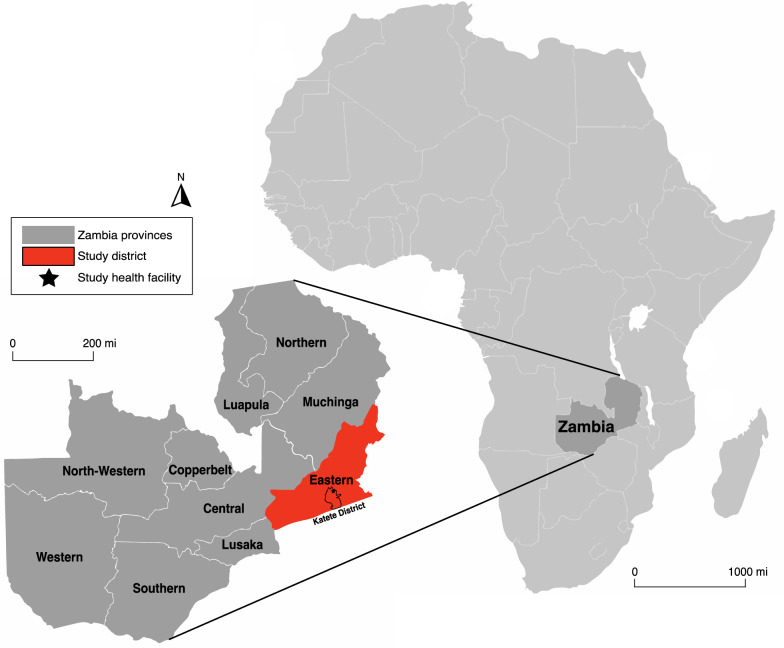
Map of the health facility location in Katete District, Eastern province, Zambia. This map was created using qGIS (v. 3.10.5) with Google images. The first map (right) shows the general area of collection in context of Africa, and the second map (left) zooms in on the country of Zambia, showing its provinces and displaying the study location

This study utilized air-dried blood spots (DBS) collected on Whatman Grade 3 filter paper (Sigma-Aldrich, St. Louis, MO, USA) from a larger cross-sectional study performed during the routine AL therapeutic efficacy studies (TES) completed between April-June 2012 and 2016 in Katete District, Eastern Zambia [19]. TES is an in vivo routine study conducted by the Zambia National Malaria Elimination Centre (NMEC) bi-annually in different selected study sites at public primary health care facilities in Zambia to assess the efficacy of first-line treatment drugs that are currently in use for treatment of malaria. DBS and blood slides are collected from every patient who visits the clinic during the period of the study. At the end of every such study, the samples are de-identified and stored at -20^o^ C for use in any further malaria research as required. The current work was conducted on archived samples collected during pre-TES enrolment in 2012 and from day-0 of individuals enrolled in the 2016 TES at a primary health facility in Katete District (Fig. 1).

All individuals with a positive blood smear with parasitaemia of 2000 parasites/µl and above were included in the TES study. This present analysis was conducted on all the available DBS samples collected in 2012 during screening, which included both negative and positive blood slide results. For the 2016 arm of the analysis, only microscopy-positive samples from enrolled individuals were available for this study, yielding a total of 241 samples.

### DNA extraction

*Plasmodium falciparum* DNA was extracted from DBS using the Chelex method. Briefly, three 3-mm punched out DBS were incubated in 1 mL of 1% saponin in phosphate buffered saline (PBS) at room temperature for 10 min. The DBS were then centrifuged at 14,000 rpm for 2 min and the supernatant was discarded. A 1-mL volume of PBS was added to the tube containing the DBS and centrifuged at 14,000 rpm for 2 min and the supernatant discarded. A volume of 150 µl nuclease-free water and 50 µl of 20% chelex suspension in nuclease-free water were then added to the tube containing the DBS filter paper and boiled at 99 °C for 10 min. Finally, samples were centrifuged at 14,000 rpm for 1 min and 100 µl of the supernatant containing extracted DNA was stored at − 20 °C until utilized for PCR.

### Amplification and detection of the *Pfcrt* K76T gene

To amplify the *Pfcrt* gene, a nested PCR was performed according to Djimde et al. using flanking primers (CRTP1 5-CCGTTAATAATAAATACACGCAG-3 and CRTP2 5-CGGATGTTACAAAACTATAGTTACC-3) as the first-round primers, which span 537 base pairs of the *Pfcrt* genes [8]. In summary, the PCR 25-µl reaction contained 12.5 µl of 2X PCR master mix (0.05 u/µl Taq polymerase, reaction buffer, 4 mM MgCl_2_ and 0.4 mM of each dNTP) from Thermo Fisher Scientific (Waltham, MA, USA) 0.2 mM of each primer, and 4 µl of chelex extracted DNA. The amplification was performed under the following conditions: a 3-min initial denaturation step at 94 °C, followed by 45 cycles of 94 °C for 30 s, 56 °C for 30 s and 65 °C for 1 min, and lastly a single 3-min extension step at 65 °C. This was followed by a nested round using internal primers (CRTD1 5-TGTGCTCATGTGTTTAAACTT-3′ and CRTD2 5-CAAAACTATAGTTACCAATTTTG-3) in a 25 µl reaction. The reaction contained 12.5 µl Thermo Fisher Scientific PCR master mix, 4 µl of the template from the primary reaction was used as template. The PCR conditions for nested reaction were as follows: a 5-min initial denaturation at 95 °C, followed by 30 cycles of 92 °C for 30 s, 48 °C for 30 s and 65 °C for 30 s, and finally a single 3-min extension step 65 °C. Dd2 and 3D7 laboratory strains were used as controls for CQ resistance and CQ sensitivity, respectively. Digestion was performed using the enzyme *Apo I* (New England Biolabs, Ipswich, MA, USA). A 15-µl reaction was prepared containing 2.5 units of *Apo I* restriction enzyme and 8 µl of the nested product, the reaction was incubated at 37 °C for 12 h. The enzyme recognizes and cuts the 145 bp product form CRTD1/CRTD2 containing 76K codon but does not cut the products containing codon 76T found in CQ-resistant parasites.

### Amplification and detection of the *Pfmdr1* N86Y gene

To amplify the *Pfmdr1* gene, a nested PCR was performed using flanking primers MDR1 5′-ATGGGTAAAGAGCAGAAAGA-3′ and MDR2 5′-AACGCAAGTAATACATAAAGTCA-3′ as the first-round primers, which span 537 base pairs of the *Pfcrt* genes [20, 21]. The Taq PCR Kit NEB E5000s (New England Biolabs, Ipswich, MA. USA) (0.02 U Taq polymerase, reaction buffer, 1 mM MgCl_2_ and 0.4 mM of each dNTP), 0.2 mM of each primer, 2 µl of chelex extracted DNA. The amplification was performed under the following conditions: a 3-min initial denaturation step at 94 °C, followed by 35 cycles of 94 °C for 20 s, 49 °C for 25 s and 60 °C for 45 s, and finally a single 5-min extension step at 60 °C. This was followed by a nested round using an internal MDR3 5-TGGTAACCTCAGTATCAAAGAA-3 and flanking primer MDR 4 5-ATAAACCTAAAAAGGAACTGG-3′ in a 25 µl reaction. Dd2 and 3D7 laboratory strains were again used as controls for CQ resistance and CQ sensitivity, respectively. A 15-µl reaction was prepared containing 2.5 units of *Afl III* (New England Biolabs, Ipswich, MA. USA) restriction enzyme and 8 µl of the nested product, the reaction was incubated at 37 °C for 12 h. The enzyme recognizes and cuts the 521 bp nested PCR product containing the N86Y mutation found in CQ-resistant parasites.

### Ethics approval

Ethical approval was obtained from the University of Zambia Biomedical Research Ethics Committee (UNZABREC and ERES Converge IRB for TES 2012 and 2016, respectively). Informed consent was obtained from the participants after informing them that blood samples would be used to study malaria after the TES.

## Results

A total of 241 DBS samples were processed from Katete District, Eastern Zambia. Among these, 136 isolates were processed from 2012, of which 99 were characterized as positive by microscope slide and 37 samples were considered negative for *P. falciparum* by microscopy. Among the 105 isolates screened from the year 2016, all were microscope slide-positive samples. Among the 2012 samples processed by PCR, 112 (82.4%) and 94 (69.1%) isolates were successfully amplified for *Pfcrt* and *Pfmdr1*, respectively. Among the 2016 isolates, 69 (65.7%) and 72 (68.6%) were successfully amplified for *Pfcrt* and *Pfmdr1*, respectively.

### Baseline characteristics of the study population

Table 1 shows the demographic characteristics of the study participants associated with these samples collected in 2012 and 2016 in Eastern Province, Zambia. In total, there were 113 males (46.9%) and 128 (53.1%) females screened, with 136 individuals in 2012 and 105 individuals in 2016. The median age was 4.4 in 2012 and 7.0 in 2016.

**Table 1:** Baseline characteristics of the study population from Katete in 2012 and 2016

	Year 2012	Year 2016
Total samples	136	105
Sex distribution of individuals screened	Males 57 (41.9%)	Males 56 (53.3%)
	Females 79 (58.1%)	Females 49 (46.7%)
Median age (in years)	4.4	7.0
Mean temperatures (^o^C)	37.7	38.2
Slide positivity	99 (72.8%)	105 (100%)
Mean parasitaemia (Parasites/µl)	31,813	64,946

### Prevalence of *Pfcrt* K76T and *Pfmdr1* N86Y

**Fig. 2:**
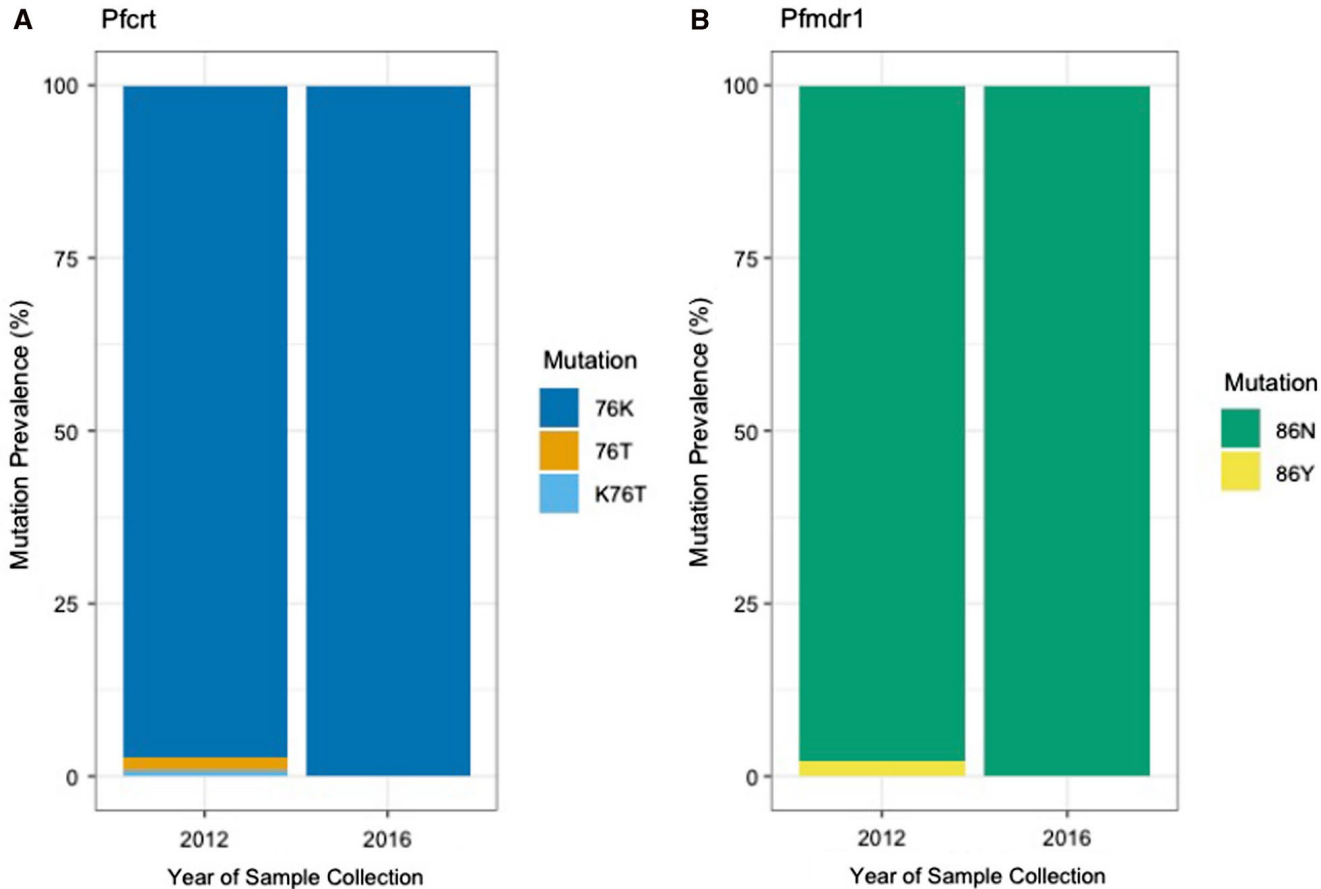
Prevalence of chloroquine resistance among collected isolates. **A** Prevalence of Pfcrt (K76T) mutations in 2012 and 2016 among samples included in study. **B** Prevalence of Pfmdr1 (N86Y) mutations in 2012 and 2016 for samples included in analysis

Overall, in 2012 the prevalence of *Pfcrt* 76K wildtype was 97.3% (109/112), followed by 1.8% for 76T mutant (2/112), and 0.9% for K76T (1/112) mixed, which contains both the sensitive and resistant markers (Table 2, Fig. 2A). For the *Pfmdr1* gene, the prevalence of 86N wildtype was 97.9% (92/94) and of 86Y mutant was 2.1% (2/94) (Table 2, Fig. 2B) in 2012.

**Table 2:** Prevalence of the *Pfcrt* 76 and *Pfmdr1* 86 mutations in Katete District, Eastern Province in 2012 and 2016

	Year 2012	Year 2016
*Pfcrt* gene	Resistant 2 (1.8%)	Resistant 0 (0%)
	Mixed 1 (0.9%)	Mixed 0 (0%)
	Sensitive 109 (97.3%)	Sensitive 69 (100%)
*Pfmdr1* gene	Resistant 2 (2.1%)	Resistant 0 (0%)
	Sensitive 92 (97.9%)	Sensitive 72 (100%)

The prevalence of *Pfcrt* 76K wildtype increased to 100% among isolates included from 2016 (69/69), with complete removal of the mutant and mixed resistance mutations (Fig. 2A). As was the case for *Pfcrt*, the prevalence of the wildtype *Pfmdr1* 86N increased to 100% among included samples from 2016 (72/72), with the complete reduction of the prevalence of the marker associated with CQ resistance (Fig. 2B). Additionally, there were samples from 2012 and 2016 in which the presence of both *Pfcrt* 76K and *Pfmdr1* 86N mutations were observed.

## Discussion

The data from the current study show a decrease in the prevalence of molecular markers associated with CQ resistance in Katete District, Eastern Zambia between 2012 and 2016, or 9 and 13 years post-CQ treatment withdrawal. Specifically, the prevalence of *Pfcrt* 76T and *Pfmdr1* 86Y both decreased in 2012 from 1.8% and 2.1%, respectively, to 0% in 2016. These data are consistent with the results from other studies conducted in Zambia, which have shown decreases in the prevalence of CQ resistance from values of 95% in 2001 to 26% in 2006 [18, 22, 23]. Specifically, one study in Zambia that examined samples for CQ-susceptible and -resistant malaria found that the prevalence of CQ-susceptible malaria increased from roughly 4% in 2001 before drug withdrawal to 79% in 2006 and 100% in 2010 [18]. Although this study was carried out in an area of high malaria transmission, in Nchelenge District, Luapula Province, it reflects the same pattern of the complete return of CQ-susceptible parasites after the removal of drug pressure. It is possible that the return of CQ-susceptibility was first observed in areas of high transmission such as Luapula Province because a large proportion of infections remain untreated, and recombination can occur during sexual reproduction in the mosquito [18]. Another study conducted in two areas of moderate and low malaria transmission, Western and Southern Provinces, respectively, showed the return of CQ-sensitive parasites 14 years after the withdrawal of the drug. These results are consistent with another report from an area of moderate malaria transmission, Ndola in Copperbelt Province, Zambia in 2017, where there was no evidence of *Pfcrt* K76T resistance markers in screened samples [24]. While there are limited consistent data for identical years from all provinces in Zambia especially in Eastern Province, this study provides data in Katete district by comparing prevalence of CQ resistance from isolates in 2012 and 2016. These findings of re-emergence of CQ-sensitive parasites or ‘chemo-reversion’, suggest that the withdrawal of anti-malarial drug pressure from the parasite population likely resulted in the re-expansion of the wild type parasites carrying the K76 and N86 codons on the *Pfcrt* and *Pfmdr1* genes, respectively [25].

Malawi, Zambia and Zimbabwe are three African countries that have reported 100% prevalence of wild-type codon K76 carrying *P. falciparum* parasites after official cessation of CQ use, additionally, a general trend towards restoration of CQ sensitivity has been reported for the continent [3, 26, 27]. Malawi was the first country to report the return of CQ-susceptible *P. falciparum* parasites 9 years after the withdrawal of CQ as treatment [14]. The return of CQ-susceptible parasites must have resulted from the re-expansion of CQ-sensitive parasites that had a survival fitness advantage over the CQ-resistant strains [25].

Kenya reported that the frequency of the *Pfcrt* K76T mutation had decreased from 95 to 23% two decades post-CQ treatment withdrawal [28]. In Rwanda, the prevalence of wild type *Pfcrt* K76 was reported to be at 50% around 14 years after cessation of CQ use, while Tanzania reported a similar K76 allelic prevalence between 85.5 to 93% 10 years after the discontinuation of CQ from treatment guidelines with regional variabilities [29, 30].

The trends in the re-emergence of CQ-susceptible genotypes seen in Zambia, Malawi and Zimbabwe can be attributed to the fact that CQ was completely withdrawn as treatment for uncomplicated falciparum malaria. The replacement for CQ, AL, has a different mode of action on the parasite and selects for CQ-sensitive *P. falciparum* [31, 32]. Furthermore, there was no CQ drug pressure on the *P. falciparum* population, which allowed the more fit CQ-sensitive parasites to thrive. This is not the case in Southeast Asia and South America, where the parasites carrying the mutation have become fixed in the population due to continued use of chloroquine in the treatment of vivax malaria [26, 33].

Malaria epidemiology may also have contributed to the re-emergence of CQ-susceptible *P. falciparum*. Malaria transmission intensity, which impacts both human host immunity and the rate of parasite recombination in the arthropod vector, contributes to the spread of the *Plasmodium* parasite. Different models have shown that high malaria transmission intensity results in high host immunity due to repeated exposure to malaria infections. Additionally, there is a high rate of parasite recombination in high transmission areas when compared to low transmission areas due to multiplicity of infections. Eastern Province was classified a high malaria transmission area at the time when the samples were collected. Therefore, the CQ-sensitive parasites, which already have a high fitness advantage over the resistant parasites, will quickly increase in the population once the drug pressure is removed. In low transmission areas, however, there are usually unique parasite population characteristics, and each individual receives one infectious bite with a single genotype. In such cases, this single genotype is taken up in the blood meal by an *Anopheles* mosquito. Consequently, during the sexual reproductive stage of the parasite in the mosquito midgut, there is little opportunity for genetic recombination resulting in a single fixed genotype in the parasite population [34].

These results should be interpreted with caution, keeping in mind the following limitations. First, the sample size for 2016 was smaller than that from 2012, which may have introduced a selection bias. More studies should be conducted with a larger sample size to acquire more accurate estimates of prevalence. Secondly, this study was conducted in one province in Eastern Zambia, so the results cannot be generalized to the whole country because of differences in epidemiologic patterns in the countries. As such, prevalence cannot be compared between provinces, so further studies should examine potential differences between areas of low and high transmission status with appropriate sampling strategies. Finally, not all of the samples that were considered positive by microscopy were also amplified by PCR, so there was a possibility of amplification bias. In particular, there was less amplification by PCR in the samples from 2016 than 2012, which could be due to individual handling or storage of the samples, and could lead to an under- or over-estimation of the prevalence.

## Conclusion

The withdrawal of CQ from use as first-line drug has resulted in the recovery of CQ-sensitive parasites in Katete District, Zambia. This observed recovery of sensitive strains seems to have occurred over a long period of time as seen in the prevalence of resistant strains in the isolates collected in 2012. This study had limitations due to fewer *P. falciparum* isolates that successfully amplified and analysed in 2016 as compared to the number of isolates from 2012. Despite this, the results obtained from this study agree with previously published data showing the recovery of CQ-sensitive strains in the parasite population. As CQ-resistant alleles were reduced completely by 2016 based on the analysed samples, this could indicate an opportunity to re-introduce CQ for malaria prevention, especially in vulnerable populations, such as in pregnant women. Additionally, this study suggests that routine national surveillance of resistance markers should be implemented at larger levels as the country continues toward achieve malaria elimination.

## Data Availability

The datasets acquired and analysed for this study will be available by request from the corresponding author.

## Abbreviations

CQ: Chloroquine
ACT: Artemisinin-based combination therapy
AL: Artemether–lumefantrine
PBS: Phosphate buffered saline
PCR: Polymerase chain reaction
*Pfcrt*: *Plasmodium falciparum* Chloroquine resistant transporter gene
*Pfmdr1*: *P. falciparum* Multi drug resistant gene 1
RFLP: Restriction fragment length polymorphism
RDT: Rapid diagnostic test
TES: Therapeutic efficacy studies
WHO: World Health Organization

## References

[1] Kamuliwo M, Chanda E, Haque U, Mwanza-Ingwe M, Sikaala C, Katebe-Sakala C (2013). The changing burden of malaria and association with vector control interventions in Zambia using district-level surveillance data, 2006–2011. Malar J.

[2] Inambao AB, Kumar R, Hamainza B, Makasa M, Nielsen CF (2017). Malaria incidence in Zambia, 2013 to 2015: observations from the health management information system. Health Press Zambia Bull.

[3] Ministry of Health. National Malaria Control Programme Strategic Plan For FY 2011–2015. Lusaka: National Malaria Elimination Centre; 2011. [https://extranet.who.int/countryplanningcycles/sites/default/files/planning_cycle_repository/zambia/zambia_malaria_nsp_2011-2015_.pdf]

[4] Ministry of Health. National Malaria Elimination Strategic Plan 2017–2021. Lusaka: National Malaria Elimination Centre; 2017. [https://static1.squarespace.com/static/58d002f017bffcf99fe21889/t/5b28d7f1575d1ff0942dbce1/1529403401067/National+Malaria+Elimination+Strategic+Plan+2017-Final_PRINT.pdf]

[5] Masaninga F, Chanda E, Chanda-Kapata P, Hamainza B, Masendu HT, Kamuliwo M (2013). Review of the malaria epidemiology and trends in Zambia. Asian Pac J Trop Biomed.

[6] Sipilanyambe N, Simon JL, Chanda P, Olumese P, Snow RW, Hamer DH (2008). From chloroquine to artemether-lumefantrine: the process of drug policy change in Zambia. Malar J.

[7] WHO. Guidelines for the treatment of malaria. 3rd ed. Geneva: World Health Organization; 2015. 26020088

[8] Djimdé A, Doumbo OK, Cortese JF, Kayentao K, Doumbo S, Diourté Y (2001). A molecular marker for chloroquine-resistant *falciparum* malaria. N Engl J Med.

[9] Reed MB, Saliba KJ, Caruana SR, Kirk K, Cowman AF (2000). Pgh1 modulates sensitivity and resistance to multiple antimalarials in Plasmodium falciparum. Nature.

[10] Setthaudom C, Tan-ariya P, Sitthichot N, Khositnithikul R, Suwandittakul N, Leelayoova S (2011). Role of Plasmodium falciparum chloroquine resistance transporter and multidrug resistance 1 genes on in vitro chloroquine resistance in isolates of Plasmodium falciparum from Thailand. Am J Trop Med Hyg.

[11] Zakeri S, Afsharpad M, Kazemzadeh T, Mehdizadeh K, Shabani A, Djadid ND (2008). Association of pfcrt but not pfmdr1 alleles with chloroquine resistance in Iranian isolates of Plasmodium falciparum. Am J Trop Med Hyg.

[12] Dorn A, Vippagunta SR, Matile H, Jaquet C, Vennerstrom JL, Ridley RG (1998). An assessment of drug-haematin binding as a mechanism for inhibition of haematin polymerisation by quinoline antimalarials. Biochem Pharmacol.

[13] Laufer MK, Thesing PC, Eddington ND, Masonga R, Dzinjalamala FK, Takala SL (2006). Return of chloroquine antimalarial efficacy in Malawi. N Engl J Med.

[14] Kublin JG, Cortese JF, Njunju EM, Mukadam RA, Wirima JJ, Kazembe PN (2003). Reemergence of chloroquine-sensitive Plasmodium falciparum malaria after cessation of chloroquine use in Malawi. J Infect Dis.

[15] Kiarie WC, Wangai L, Agola E, Kimani FT, Hungu C (2015). Chloroquine sensitivity: diminished prevalence of chloroquine-resistant gene marker pfcrt-76 13 years after cessation of chloroquine use in Msambweni. Kenya Malar J.

[16] Mekonnen SK, Aseffa A, Berhe N, Teklehaymanot T, Clouse RM, Gebru T (2014). Return of chloroquine-sensitive Plasmodium falciparum parasites and emergence of chloroquine-resistant Plasmodium vivax in Ethiopia. Malar J.

[17] Bogreau H, Renaud F, Bouchiba H, Durand P, Assi SB, Henry MC (2006). Genetic diversity and structure of African Plasmodium falciparum populations in urban and rural areas. Am J Trop Med Hyg.

[18] Mwanza S, Joshi S, Nambozi M, Chileshe J, Malunga P, Kabuya J-BB, et al. The return of chloroquine-susceptible *Plasmodium falciparum* malaria in Zambia. Malar J. 2016;15:584. 10.1186/s12936-016-1637-3 PMC5139104 27919256

[19] Hamainza B, Masaninga F, Moonga H, Mwenda M, Chanda-Kapata P, Chalwe V (2014). Therapeutic efficacy of artemether-lumefantrine on treatment of uncomplicated Plasmodium falciparum mono-infection in an area of high malaria transmission in Zambia. Malar J.

[20] Wang X, Mu J, Li G, Chen P, Guo X, Fu L, et al. Decreased prevalence of the *Plasmodium falciparum* chloroquine resistance transporter *76T* marker associated with cessation of chloroquine use against *P. falciparum* malaria in Hainan, People's Republic of China. Am J Trop Med Hyg. 2005;72:410–4. 15827277

[21] Antony HA, Das S, Parija SC, Padhi S (2016). Sequence analysis of pfcrt and pfmdr1 genes and its association with chloroquine resistance in Southeast Indian Plasmodium falciparum isolates. Genom Data.

[22] Sitali L, Mwenda MC, Miller JM, Bridges DJ, Hawela MB, Chizema-Kawesha E (2019). En-route to the ‘elimination’ of genotypic chloroquine resistance in Western and Southern Zambia, 14 years after chloroquine withdrawal. Malar J.

[23] Sagara I, Oduro AR, Mulenga M, Dieng Y, Ogutu B, Tiono AB (2014). Efficacy and safety of a combination of azithromycin and chloroquine for the treatment of uncomplicated Plasmodium falciparum malaria in two multi-country randomised clinical trials in African adults. Malar J.

[24] Kasonde-Chanshika B, Shimaponda-Mataa NM. Profiling chloroquine resistance-associated *Pfcrt-76T* and *Pfmdr1–86Y* mutations in *Plasmodium falciparum* isolates of Ndola, Zambia. Zambia National Health Conference. Lusaka: NHRA. 2018; 204.

[25] Laufer MK, Takala-Harrison S, Dzinjalamala FK, Stine OC, Taylor TE, Plowe CV (2010). Return of chloroquine-susceptible falciparum malaria in Malawi was a reexpansion of diverse susceptible parasites. J Infect Dis.

[26] Ocan M, Akena D, Nsobya S, Kamya MR, Senono R, Kinengyere AA, Obuku EA (2019). Persistence of chloroquine resistance alleles in malaria endemic countries: a systematic review of burden and risk factors. Malar J.

[27] Lu F, Zhang M, Culleton RL, Xu S, Tang J, Zhou H (2017). Return of chloroquine sensitivity to Africa? Surveillance of African *Plasmodium falciparum* chloroquine resistance through malaria imported to China. Parasit Vectors.

[28] Kishoyian G, Njagi ENM, Orinda G, Kimani F (2018). Chloroquine sensitivity and prevalence of chloroquine-resistant genes pfcrt and pfmdr-1 in Western Kenya after two decades of chloroquine withdrawal. Ann Med Health Sci Res.

[29] Mohammed A, Ndaro A, Kalinga A, Manjurano A, Mosha JF, Mosha DF (2013). Trends in chloroquine resistance marker, Pfcrt-K76T mutation ten years after chloroquine withdrawal in Tanzania. Malar J.

[30] Kateera F, Nsobya SL, Tukwasibwe S, Hakizimana E, Mutesa L, Mens PF (2016). Molecular surveillance of Plasmodium falciparum drug resistance markers reveals partial recovery of chloroquine susceptibility but sustained sulfadoxine-pyrimethamine resistance at two sites of different malaria transmission intensities in Rwanda. Acta Trop.

[31] Baraka V, Tinto H, Valea I, Fitzhenry R, Delgado-Ratto C, Mbonye MK (2015). In vivo selection of Plasmodium falciparum Pfcrt and Pfmdr1 variants by artemether-lumefantrine and dihydroartemisinin-piperaquine in Burkina Faso. Antimicrob Agents Chemother.

[32] Sisowath C, Petersen I, Veiga MI, Mårtensson A, Premji Z, Björkman A (2009). In vivo selection of Plasmodium falciparum parasites carrying the chloroquine-susceptible pfcrt K76 allele after treatment with artemether-lumefantrine in Africa. J Infect Dis.

[33] Alam MS, Ley B, Nima MK, Johora FT, Hossain ME, Thriemer K (2017). Molecular analysis demonstrates high prevalence of chloroquine resistance but no evidence of artemisinin resistance in Plasmodium falciparum in the Chittagong Hill Tracts of Bangladesh. Malar J.

[34] Takala-Harrison S, Laufer MK (2015). Antimalarial drug resistance in Africa: key lessons for the future. Ann N Y Acad Sci.

